# Prevalence of Undiagnosed Depression among Persons with Hypertension and Associated Risk Factors: A Cross-Sectional Study in Urban Nepal

**DOI:** 10.1371/journal.pone.0117329

**Published:** 2015-02-11

**Authors:** Dinesh Neupane, Bindu Panthi, Craig S. McLachlan, Shiva Raj Mishra, Brandon A. Kohrt, Per Kallestrup

**Affiliations:** 1 Center for Global Health, Department of Public Health, Aarhus University, Aarhus, Denmark; 2 Nobel College, Faculty of Science and Technology, Kathmandu, Nepal; 3 Rural Clinical School, University of New South Wales, Sydney, Australia; 4 Shiva Raj Mishra, Nepal Development Society, Kathmandu, Nepal; 5 Brandon A. Kohrt, Duke Global Health Institute, Duke University, Durham, North Carolina, United States of America; University of Tolima, COLOMBIA

## Abstract

**Background:**

Despite an increasing number of studies exploring prevalence of depression among hypertensive patients in high income countries, limited data is available from low and middle income countries, particularly Nepal. Our aim was to investigate the prevalence of undiagnosed (sub clinical) depression and associated risk factors among hypertensive patients attending a tertiary health care clinic in Nepal.

**Methods:**

The study was based on a cross-sectional study design, with 321 hypertensive patients attending the Out-Patient Department of a central hospital in Nepal. Blood measure was recorded via a mercury column sphygmomanometer. Depression levels were assessed using the Beck Depression Inventory-Ia (BDI) scale. Demographics and risk factors were assessed.

**Result:**

The proportion of participants with undiagnosed depression was 15%. Multivariable analyses demonstrated an *increase in BDI scores with increased aging*. Approximately a 1 point increase in the BDI score was observed for *each additional decade of aging in hypertensive patients*. Additional factors associated with increased risk of depression included being female (4.28 point BDI score increase), smoking (5.61 point BDI score increase), being hypertensive with no hypertensive medication (4.46 point BDI score increase) and being illiterate (4.46 point BDI score increase).

**Conclusions:**

Among persons with hypertension in outpatient settings in Nepal, demographic (age, sex, education), behavioural (smoking,) and adherence factors (anti-hypertensive medication) were associated with undiagnosed depression. Screening programs in Nepal may assist early intervention in hypertensive patients with sub clinical depression.

## Introduction

Both hypertension and depression emerge from a complex interaction of social, behavioural and biological factors [1, 2]. Depression is common in patients with chronic diseases including hypertension. Sub clinical depression may evade diagnosis in these patients [3]. When both co morbidities exist (sub clinical depression and hypertension), there is increased risk for reduced quality of life, increased risk of stroke or myocardial infarction, reduced therapeutic compliance for anti-hypertensive therapy, increased risk of suicide in future, developed uni polar depression and higher use of limited health care resources [4, 5, 6, 7]. Epidemiological studies performed in high income countries have consistently demonstrated an increased co-occurrence of depression with hypertension [8]. Despite an increasing number of studies exploring the prevalence of depression among hypertensive patients in high income countries, limited data is available from Nepal and other low and middle income countries. Community-based epidemiological studies in Nepal suggest that the prevalence of depression ranges from 28% to 41% [9, 10] and prevalence of hypertension ranges from 23% to 34% [11–15]. Despite the high prevalence of depression and hypertension in Nepal, no study to date has been specifically designed to explore the interactions between hypertension and depression. Health data collected on the prevalence of comorbid hypertension and sub clinical depression in Nepal is useful as it will allow for health care planning.

The aim of this study is to explore the prevalence of undiagnosed depression among hypertensive patients attending an Out-Patient Department (OPD) of the National Heart Centre in Kathmandu in Nepal.

## Material and Methods

### Ethical Approval

Eligible participants provided written informed consent. The study was conducted in accordance with the Declaration of Helsinki and local ethical approval was obtained from the Institutional Review Board of Nobel College, Pokhara University. Permission for carrying out the research was also obtained from the Hospital administration.

### Study Design

A cross-sectional study was conducted during the two months from the 5th of August to the 4th of October 2011 at the OPD of the Shahid Gangalal National Heart Centre (SGNHC) in Kathmandu, Nepal. This is the largest tertiary level hospital for cardiovascular care in Nepal. In the OPD, there was a registration of the patient before the examination, between 9:00 to 11:00 AM including hypertensive status of the patient. The Health Management Information System (HMIS) of the Government of Nepal uses an ethnicity classification system with six categories, namely, (i) Dalits, (ii) Disadvantaged Janajatis, (iii) Disadvantaged Non-Dalit Terai Caste Groups, (iv) Religious Minorities, (v) Relatively Advantaged Janajatis, and (vi) Upper Caste Groups [16]. Based on the data available, we collapsed ethnicity sub-groups into three groups: (i) Upper Caste (ii) Janajati (iii) Other.

### Study Parameters

**Demographic data**. Participants were interviewed and information regarding age, sex, ethnicity, family type, education, monthly income, co-morbidity, family history of hypertension, physical activity, diet and life style factors (e.g. smoking and alcohol intake) were collected.

**Anthropometry**. Height and weight measured. The Body Mass Index (BMI) calculated as Kg/m^2^ and categorized as normal (<25 Kg/m^2^) overweight (25–29.9 Kg/m^2^) and obese (>29.9 kg/m^2^) according to WHO classifications [17].

**Blood pressure**. Hypertension defined as systolic blood pressure (SBP) ≥140 mmHg and/or diastolic blood pressure (DBP) ≥90 mmHg or taking anti-hypertensive drugs or previously diagnosed by health care workers. All the participants in the cohort were defined as hypertensive. For the purpose of measuring blood pressure, the Seventh Report on Joint National Committee on Prevention, Detection, Evaluation, and Treatment of High Blood Pressure (JNC VII) guideline was followed for measuring blood pressure [18], using a standard mercury column sphygmomanometer.

**Depression**. Depression assessments were made via *Beck Depression Inventory* (BDI-Ia) [19]. The BDI-Ia is a scale of 21 items with four response options, which assesses and quantifies the number of depressive symptoms across broad domains such as sadness, hopelessness, feeling of guilt, changes in sleep, and appetite. Patients will be asked to recall events from the week before the interview. Thus the 21-item BDI-Ia assesses depression symptoms over the prior two weeks. Items are scored 0–3 with an instrument range of 0 to 62. The BDI is validated for use in Nepal [20], for example with a clinical DSM-IV diagnoses of major depressive disorder (MDD): area under the curve (AUC) 0.92 (95% CI 0.88–0.96); internal reliability (Cronbach alpha), α = 0.90. Test-retest reliability for the BDI was 0.84 using a Spearman-Brown coefficient. Based on the clinical validation of the BDI in Nepal, a score of 20 or higher suggests moderate depression symptoms with the need for mental health intervention (sensitivity = 0.73, specificity = 0.91) [20]. Therefore in this study, we refer BDI ≥20 as “depression” and BDI <20 as “non depression” in categorical analyses.

**Major modifiable risk factors for hypertension**. Smoking, alcohol, low physical activity and unhealthy diet were classified according to WHO classification [21]. ***Smoking***: Participants who smoked any form of tobacco products on a daily basis at the time of data collection were included as smokers. ***Nutrition***: Subjects consuming less than ≤2 servings of fruits or vegetables daily were classified as having a low intake of fruits and vegetables. ***Physical activity***: Individuals performing physical activity less than 150 minutes per week were classified as having low physical activity. ***Alcohol consumption***: it was recorded as the frequency of intake and quantity of spirits, beer and wine per week. These data were converted into standard drink/s (1 standard drink = 250 ml beer, 30 ml = local spirit and hard liquor; 105 ml = wine). For men ≥ 5 standard drinks and for women ≥ 4 standard drinks were classified as heavy episodic drinking [22].

**Literacy level**. Literacy level was characterised as literate (Can read and write) or illiterate (Cannot read and write).

**Monthly income**. Monthly income of household was first recorded in Nepali Rupees and converted to US Dollar (100 NPR = 1 USD).

### Exclusion and Inclusion Criteria

Only outpatients above 25 years of age and previously diagnosed with hypertension for a duration of at least 6 months were included in the study. Patients with complications such as hypertensive retinopathy, nephropathy, encephalopathy and women who were pregnant were excluded from the study. Patients with a prior history of clinically diagnosed depression or currently taking anti-depressant medication were excluded.

### Statistical Analysis

Epi Data 3.1 was used for the data entry and STATA 13 was used for data analysis. Descriptive analyses were presented in frequency and percentages. Statistical comparisons of categorical variables were conducted using chi-square tests. For the purpose of exploring association between BDI-Ia scores and other independent variables, linear regression analysis was used. Since the BDI-1a score did not follow normality assumptions and log transformation models remained inconclusive, bootstrapping regression models were used with 50 replications. Bivariate and multivariate analyses were conducted for effect estimation. Sex, age, smoking, diabetes, antihypertensive medicine and literacy status were adjusted in the regression analysis because they were found statistically significant in bivariate analysis. P-values <0.05 were considered statistically significant.

## Results

### Sociodemographic and basic clinical characteristics of participants by depression category

Socio-demographics and basic clinical characteristics of participants are presented in Table 1. The proportion of male and female participants was roughly equal. Sixty percent of participants (60%) belonged to Upper Caste Groups and 22% of the participants were illiterate. The mean age was 52.70 ± 13.30 years and the mean Body Mass Index (BMI) of the participants was 25.43 ± 3.83 kg/m^2^. The mean systolic and diastolic blood pressures were 146.31 ± 19.73 mmHg and 91.24 ± 11.99 mmHg respectively. The average period of diagnosed hypertension among the participants was 5 ± 5.16 years. The average monthly income of participant’s household was US$ 261 ± 408.

**Table 1:** Sociodemographic and basic clinical characteristics of participants by depression status.

Variable	Total	No Depression (BDI<20)	Depression (BDI> = 20)	p-value
Sex				
Male	170(53%)	155(91%)	15(9%)	0.001
Female	151(47%)	118(78%)	33(22%)	
Ethnicity				
Uppercaste	194(60%)	164(85%)	30(15%)	0.243
Janjati	103(32%)	91(88%)	12(12%)	
Others	24(8%)	18(75%)	6(25%)	
Educational status				
Illiterate	70(22%)	47(67%)	23(33%)	<0.001
Literate	251(78%)	226(90%)	25(10%)	
Age (Years)				
25–44	82(26%)	73(89%)	9(11%)	<0.001
45–64	169(53%)	150(89%)	19(11%)	
>64	70(22%)	50(71%)	20(29%)	
Body Mass Index (kg/m^2^)				
Normal (<25)	157(49%)	135(86%)	22(14%)	0.042
Overweight (25–29.9)	125(39%)	110(88%)	15(12%)	
Obese (>29.9)	39(12%)	28(72%)	11(28%)	
Monthly income(US$)				
Less than US$ 260	256(80%)	216(84%)	40(16%)	0.503
More than US$ 260	65(20%)	57(88%)	8(12%)	
Systolic Blood Pressure (mm Hg)				
<140	102(32%)	82(80%)	20(20%)	0.111
≥140	219(68%)	191(87%)	28(13%)	
Diastolic Blood Pressure (mm Hg)				
<90	105(33%)	90(86%)	15(14%)	0.815
≥90	216(67%)	183(85%)	33(15%)	

### Depression

The mean BDI-1a score for all participants was 8.55 ± 10.79. Male and female scores were 6.27 ± 9.23 and 11.11 ± 11.83 respectively. The proportion of participants with undiagnosed depression (BDI-1a score ≥20) was 15%. Statistical differences (p<0.05) were observed for sex, literacy status, age and BMI when adjusted for depression or non-depression sub- groups. Females had a 22% prevalence rate for depression compared to males who had only 9%. Illiterate participants had a 33% prevalence rate for depression compared to literate participants who had only 10%. The prevalence of depression among age sub-groups 25–44 years, 45–64 years and more than 64 years were 11%, 11% and 29% respectively. The highest prevalence rates for depression were observed among obese (BMI > 29.9 kg/m^2^) participants (28%). A trend was observed for lower average systolic and diastolic blood pressure, higher BMI, and a longer duration of clinically diagnosed hypertension in participants with depression, although the results did not reach statistical significance.

### Major risk factors of hypertension and co-morbidities of participants by depression status

The health behaviours and co-morbidities of participants stratified on the basis of depression status are presented in Table 2. The only statistically significant modifiable external factors in depression were a lower intake of fruit and green vegetables (p = 0.017) and smoking (p<0.01).

**Table 2:** Health behaviours and co-morbidities of participants by depression status.

Variable	Total	No Depression (BDI<20)	Depression (BDI> = 20)	p-value
Physical Activity(n = 251)				
< 150 minute/week	32(13%)	29(91%)	3(9%)	0.777
≥150 minute/week	219(87%)	192(88%)	27(12%)	
Fruit and green vegetables				
<2 serving/day	50(16%)	37(74%)	13(26%)	0.017
≥ 2 serving/day	271(84%)	236(87%)	35(13%)	
Smoking				
Yes	37(11%)	26(70%)	11(30%)	0.007
No	284(88%)	247(87%)	37(13%)	
Heavy Episodic Drinking of Alcohol*				
Yes	18(6%)	16(89%)	2(11%)	0.990
No	303(94%)	257(85%)	46(15%)	
Diabetes				
Yes	63(20%)	49(78%)	14(22%)	0.071
No	258(80%)	224(87%)	34(13%)	
High Cholesterol				
Yes	60(19%)	54(90%)	6(10%)	0.233
No	261(81%)	219(84%)	42(16%)	
Antihypertensive medication				
Yes	267(83%)	230(86%)	37(14%)	0.221
No	54(17%)	43(80%)	11(20%)	
Family history of hypertension				
Yes	103(32%)	89(86%)	14(14%)	0.638
No	218(68%)	184(84%)	34(16%)	

*Heavy Episodic Drinking of Alcohol = > 3 standard drink for women and >4 standard drink for men

Bivariate regression analysis presented in Table 3 showed that being female (β = 4.83, 95% CI: 2.61; 7.05), older age (β = 0.11, 95% CI: 0.01; 0.22), smoking (β = 5.18; 95% CI: 0.39; 9.96), diabetes (β = 3.57; 95% CI: 0.62; 6.53), and being illiterate (β = 6.49; 95% CI: 3.70; 9.27) were associated with a higher BDI score. We also observed that taking anti-hypertensive medication (β = -2.89; 95% CI: -6.85; -1.53) was significantly associated with a lower BDI score.

**Table 3:** Bivariate Regression Analysis between Depression Score and Major Risk Factors of Hypertension.

Non-Modifiable Risk Factors	Coefficient (95% CI)
Age (Year)	0.11(0.01; 0.22)
Sex (Female)	4.83(2.61; 7.05)
Ethnicity (Upper caste)	-0.22(-2.05; 1.61)
Parental hypertension (Yes)	-0.03(-2.50; 2.42)
Modifiable Risk Factors	
Smoking (current smoker)	5.18(0.39; 9.96)
Alcohol (Heavy episodic drinking)	0.18(-6.02; 6.39)
Physical activity (<150 min/wk)	-2.63(-6.07; 0.80)
Unhealthy diet (<2 serving)	1.0(-1.93; 3.94)
Metabolic Risk Factors	
Systolic Blood Pressure (mmHg)	-0.024(-0.08; 0.03)
Diastolic Blood Pressure (mmHg)	-0.009(-0.10; 0.08)
Diabetes (Yes)	3.57(0.62; 6.53)
Body Mass Index (Kg/m2)	-0.92(-0.38; 0.20)
Anti-hypertensive medication (Yes)	-2.89(-6.85; -1.05)
High Cholesterol (Yes)	-0.944(-3.58; 1.69)
Other socio-economic variable	
Education (Illiterate)	6.49(3.70; 9.27)
Income	-1.250(-3.870; 1.360)
Ethnicity (Upper caste)	-0.430(-0.226; 0.140)

Multivariate regression analysis in Table 4 revealed similar results except for diabetes. Interestingly, multivariable analyses showed that for each decade (10-year) increase in age this was associated with a 1 point increase in BDI-1a scores. Other associated variables included being female (4.28 point BDI score increase), smoking (5.61 point BDI score increase) and taking no hypertensive medication (4.21 point BDI score increase).

**Table 4:** Multivariate Regression Analysis.

Variable	Coefficient (95% CI)**
Sex (Female)	4.28(1.85; 6.72)
Age	0.10(0.01; 0.19)
Smoking (Yes)	5.6(0.82; 10.54)
Diabetes (Yes)	2.85(-0.49; 6.20)
Education (Literate)	-4.46(-8.19; -0.74)
Antihypertensive Medicine (Yes)	-4.21(-7.98; -0.44)

**Adjusted for age, sex, smoking, diabetes and education

## Discussion

This is the first cross-sectional study from Nepal to document the prevalence of undiagnosed depression among hypertensive patients. The prevalence of undiagnosed depression in our hypertensive population is lower than that observed in other populations in Nepal. For example, using a similar BDI cut-off ≥20, the prevalence of undiagnosed depression among patients with diabetes in outpatient settings in Kathmandu is 40% [23]. The prevalence in our study is also lower than that observed among persons living with HIV/AIDS in Kathmandu [24]. Among community settings in rural north western Nepal the depression prevalence rate has been documented to be as high as 41% [25], and in southern Nepal, 17–43% [26]. Our study also had a lower prevalence rate of undiagnosed depression compared to studies in diabetic patients where hypertension would also be prevalent [23].

From our regression analysis, it was found that age, sex, smoking, education and antihypertensive medication were associated with higher BDI score. We also found that smoking was a strong predictor of depression. Indeed there is evidence to suggest that nicotine dependence may lead to an increased risk of depression [33]. Similar to our study, other community-based studies have found that increasing age is also a predictor of depression in Nepal [23, 25, 26, 27]. A clear understanding of why there is a higher prevalence of depression among females as compared to males remains unknown. Weaver and Hadely [28] found in female diabetics in India an increased risk for depression if women were not fulfilling gender-specific social roles such as getting up early, preparing family meals, looking after children and grandchildren, cleaning the house, running errands. In Nepal it is not uncommon for women to be left in the home alone to tend to both home duties and the farm [29]. In addition to workforce factors, there are also physiological and genetic factors leading to a higher risk for depression in females [30,31]. It has been suggested that genetic depression risk factors overlap with cardiovascular (hypertension) risk factors, particularly in women [32].

Importantly we have demonstrated that taking anti-hypertensive medicine is significantly associated with a decrease in BDI scores. Though we do not have information on which type of anti-hypertensive medicines were used by our participants, another study conducted in the same hospital showed that the most common antihypertensive agents were Calcium Channel Blockers (65%) followed by Beta Blockers (52%) [34]. In terms of beta-blockers, Atenolol was the most common beta blocker prescribed; Propranolol was not [34]. Previous studies have failed to identify an association between depressive symptoms and Calcium Channel Blockers [35–37]. Only Propranolol has been reported to be associated with depression in clinical trial adverse event reports [38]. These *anecdotal reports* have led to a long-held belief that highly lipophilic beta blockers (such as Propranolol) are more likely than hydrophilic beta blockers such as Atenolol to be associated with clinical depression. On the other hand Bright et al demonstrated that beta-blockers of any class were not associated with depression in a case-control study examining 4,302 New Jersey Medicaid records [39]. The low prevalence of depression among our hypertensive patients could theoretically be due to pleotropic actions of anti-hypertensive drugs. Anti-hypertensive medications have been demonstrated in previous studies to reduce systemic low grade inflammation, a risk factor for depression [40,41].

Ethnicity has been previously shown to be a risk factor for depression and hypertension in some developing countries in Asia. We found that ethnicity (caste classification) was not significantly associated with depression in our hypertensive cohort. A study conducted among diabetic patients from Nepal also reported a non-significant association with ethnicity and depression [23, 25]. However, a community-based study from rural Nepal showed a greater prevalence of depression among low-caste Dalit groups [27]. Our study population was in a higher caste system, and this was supported by our population also having a higher literacy rate, of 78%. Our findings suggest that higher caste’s may be less effected by sub clinical depression (given our lower prevalence rates) if hypertension pre-exists and is treated. Furthermore populations with a higher socio-economic background are more likely to have access to and attend tertiary health care facilities in developing countries. That is identifying and treating chronic conditions will likely have a positive impact on mental health.

We could not find a statistically significant association between heavy episodic drinking and BDI scores. Previous studies suggest an association between depression and alcohol use [42]. However, low-to-moderate total alcohol intake may reduce the incidence of depression, while heavy drinkers seem to be at higher risk for depression [43]. Indeed most of our participants consumed alcohol in low-moderate amounts, and only two participant’s consumed more than 11 standard drinks per day.

Several limitations of this study need to be considered. Due to the use of subjective self-report measures, participants may give responses that are considered socially acceptable, instead of providing actual practices. Mental health self-reports are sometimes subject to bias because of a general community stigma towards mental illness in Nepal [44]. Our study would underestimate the total prevalence of overall depression because patients with prior history of clinically diagnosed depression or those patients currently taking anti-depressant medication were excluded.

## Conclusions

The proportion of participants attending a hypertension clinic in urban Nepal with undiagnosed (sub clinical) depression was 15%. Among our hypertensive patients the following factors were associated with sub clinical depression, including demographic (age, sex, education), behavioural (smoking) and adherence factors (failure to maintain or take anti-hypertensive medications). Our findings have the following implications—(1) a suggested need for better screening and treatment of hypertension across all caste systems in Nepal; (2) a need for screening and treatment of sub clinical depression in hypertension clinics in Nepal and (3) a need for interventions that address factors associated with sub clinical depression in hypertension, such as smoking cessation programs, and improved anti-hypertension medication compliance.
